# Involvement of pharmaceutical companies at scientific meetings

**Published:** 2010-06

**Authors:** Andries Brink

**Affiliations:** Editor-in-chief, Cardiovascular Journal of Africa

South Africa not infrequently hosts high-level cardiology meetings outside the auspices of South African professional medical societies and associations. Well-known cardiologists, particularly from the USA and Europe are invited to make presentations, which bring the best and most-recent advances in the field to this country. This is of course of great value to the selected group of delegates who are invited to attend. Large sums of money are involved because all the presenters and many of those attending receive full financial support for their participation.

A recent meeting in Cape Town, Cardiology and Diabetes at the Limits, is a case in point. It was arranged through the Hatter Institute in London and, typically, had as many as 30 invited cardiologists. In this instance, organisers raised funds very successfully from international pharmaceutical companies and also from their local business units. It was held outside the orbit of accountable professional medical organisations in either South Africa or the United Kingdom. These companies are hard pressed to say no, and might well wish to spend budgeted money on other forms of continuing medical education or research support, which would reach a wider audience, but are being energetically lobbied into funding such meetings.

The question arises whether South Africa is being used to sidestep the ethical principles as set out for the promotion of pharmaceutical products by the International Federation of Pharmaceutical Manufacturers and Associations (IFPMA) and the affiliated Pharmaceutical Industry Association of South Africa (PIASA).^1^ No financial statements regarding income and expenses are ever presented by these organisers, as is the practice with professional medical associations in South Africa, who typically report back to their membership at their annual general meetings. To whom and where the money goes is not therefore in the public domain. Careful monitoring of these types of events should be the local responsibility of the South African Health Professions Council and other relevant medical professional associations.

The recent editorial article by DJ Rothman, *et al.*^2^ is instructive. Some of these guidelines are already followed by South African professional medical societies, although further rigour may well be required.
